# Analysis of urinary kidney-specific cell-free DNA in preeclampsia

**DOI:** 10.1371/journal.pone.0359105

**Published:** 2026-09-29

**Authors:** Vanessa C. Heron, John B. Whitlam, David H. Murray, Nicky Boulter, Peter Mount, Suet-Wan Choy, Marina Katerelos, Kathy Paizis

**Affiliations:** 1 Department of Nephrology, Austin Health, Heidelberg, Victoria, Australia; 2 Department of Obstetrics, Mercy Hospital for Women, Heidelberg, Victoria, Australia; 3 Department of Medicine, University of Melbourne, Parkville, Victoria, Australia; 4 Garvan Institute of Medical Research, Darlinghurst, New South Wales, Australia; 5 School of Biomedical Sciences and Pharmacy, Faculty of Health and Medicine, University of Newcastle, Callaghan, New South Wales, Australia; Mayo Clinic Rochester, UNITED STATES OF AMERICA

## Abstract

**Aim:**

To assess the feasibility of a novel methodology for quantifying kidney-specific cell-free DNA (ks-cfDNA) and explore whether levels differed between individuals with preeclampsia (PE) and normotensive pregnant controls (NP).

**Background:**

Cell-free DNA (cfDNA) is released during cell death and circulates in bodily fluids, including urine. cfDNA quantities reflect the degree of organ injury. As PE is associated with hypertension and kidney injury, we hypothesised that urinary ks-cfDNA would be elevated in PE.

**Methods:**

In this single-centre prospective case-control study urine samples were collected from 20 women with PE and 20 NP controls. Digital PCR was used to quantify three kidney-specific epigenetic targets (ks-et): ks-et1 (pan-kidney marker), ks-et2 (renal proximal tubular epithelial cell (RPTEC) and podocyte marker) and ks-et3 (RPTEC marker). The beta-actin gene (ACTB) represented total cfDNA (t-cfDNA).

**Results:**

ks-et2 and ks-et3 were higher in the PE group. Median ks-et2 was 231 copies/mL (IQR 65–383) versus 62 copies/mL (IQR 29–215) in NP controls (*P* = 0.028). Median ks-et3 was 81 copies/mL (IQR 28–199) in PE versus 20 copies/mL (IQR 9–81) for NP participants (*P* = 0.047). ks-et1 and t-cfDNA showed non-significant increases in PE. Fractional ks-et quantities normalised to t-cfDNA did not differ between groups. Urinary creatinine levels were higher in the PE group (10.9 mmol/L (IQR 4.1–15.3) versus 3.9 mmol/L (IQR 2.3–5.6), *P* = 0.005). Urine creatinine demonstrated the highest AUC (0.76, 95% CI 0.59–0.92).

**Conclusion:**

This exploratory study found that quantification of urinary ks-cfDNA was feasible and that absolute quantities of ks-cfDNA markers ks-et2 and ks-et3 were elevated in PE. Consistent with previous studies, urinary creatinine was a good predictor of PE and outperformed ks-cfDNA in discriminating between preeclamptic and normotensive pregnancies. Further studies are needed to determine the clinical utility and predictive ability of ks-cfDNA in PE.

## Introduction

Preeclampsia (PE) affects 5% of pregnant women after 20 weeks’ gestation and is characterised by new-onset hypertension and multi-organ dysfunction, including kidney involvement [1]. While proteinuria and acute kidney injury are common manifestations of the kidney injury in PE, the pathogenetic mechanisms underlying these kidney changes remain incompletely understood [2]. In women with pre-existing kidney disease and/or hypertension, diagnosing PE can be challenging. Furthermore, standard measures of kidney health (serum creatinine and urine protein-to-creatinine ratio (uPCR)) are altered in pregnancy and not predictive of clinical disease onset.

Cell-free DNA (cfDNA) is an extracellular nucleic acid present in bodily fluids including blood and urine [3]. cfDNA in the plasma is primarily derived from the demise of leukocytes [4]. A small amount of cfDNA originates from solid organs and, in cases of organ injury or damage, the quantity of cfDNA increases [5]. Cell-free DNA has a short half-life of two minutes to two hours, allowing for real-time analysis of pathological processes associated with cfDNA release [6].

In transplantation, there is an established association between donor-derived cfDNA (dd-cfDNA) released from the allograft and pathology affecting that organ [7]. Similar to antibody-mediated rejection in transplanted organs, PE is associated with glomerular endothelial injury. Unlike the transplant context, where genetic polymorphisms between individuals can be used to distinguish dd-cfDNA from recipient cfDNA, in a non-chimeric individual this is not possible. Therefore, this study used kidney-specific epigenetic markers to distinguish kidney-specific cfDNA (ks-cfDNA) from total cfDNA.

Beyond transplantation and organ injury, cfDNA has been extensively studied in pregnancy-related contexts. Fetal circulating cfDNA was first reported in 1997 and is now commonly used in the maternity setting for non-invasive prenatal diagnosis [8]. In the search for a biomarker predictive of PE in early pregnancy, cfDNA has been investigated. Total maternal circulating cfDNA has been found to be higher in women with PE compared to those with normotensive pregnancies or isolated fetal growth restriction [9]. Furthermore, a lower fetal fraction of circulating cfDNA has been reported to be predictive of PE [10].

cfDNA is also detectable in the urine. Urinary cfDNA originates from two primary sources. Firstly, circulating cfDNA is filtered into the urine by the glomeruli. Secondly, cfDNA is released directly into the urinary filtrate by kidney and urinary tract cells [11]. The nephron comprises various cell types, each with its own unique epigenetic signature [12]. The proportion of ks-cfDNA originating from each cell type is hypothesised to vary in health and disease. Urinary cfDNA has been studied as a biomarker for kidney disease [4,11,13–15].

While studies have examined the presence of fetal cfDNA in maternal urine, there is currently no published literature examining changes to ks-cfDNA in women with PE [16,17]. This exploratory study aimed to assess the feasibility of a novel methodology for quantifying urinary ks-cfDNA and the biological plausibility of its use as a biomarker, while also exploring whether levels differed between individuals with PE and normotensive pregnant controls (NP). We hypothesized that PE is associated with elevated levels of ks-cfDNA, reflecting renal injury.

## Methods

### Study design

This single-centre, prospective, case-control study was granted approval by the Mercy Health Human Research Ethics Committee (2022−018). Women were recruited from the Mercy Hospital for Women, Heidelberg, Australia between March and October 2023. Urine samples were collected from 20 women after diagnosis of PE and 20 NP controls, matched by gestational age.

Inclusion criteria were adult participants more than 20 weeks gestation giving written informed consent. PE was defined as per the 2023 Society of Obstetric Medicine of Australia and New Zealand (SOMANZ) diagnostic criteria [18]. PE with severe features was defined according to the American College of Obstetricians and Gynecologists (ACOG) criteria, as severity classification is not incorporated into the relevant SOMANZ guideline [19].

Women were excluded from the NP control group if they had pre-existing hypertension, chronic kidney disease (CKD), or known fetal growth restriction at the time of screening.

Demographic and clinical data were collected from the participant and electronic medical record.

### Kidney-specific epigenetic targets

As described by Murray et al., using data generated by Illumina Infinium HumanMethylation450 (450K) or Human MethylationEPIC (EPIC) BeadChip arrays, a human methylation atlas was constructed from a repository of blood and solid tissue samples. Using this pipeline, kidney-specific DNA methylation CpG sites were identified and filtered to three differentially methylated regions (DMRs) across three genes. These DMRs were subsequently developed into PCR targets for a multiplexed digital PCR (dPCR) assay [20].

For the present study we have referred to each gene as a kidney-specific epigenetic target (ks-et). These genes are:

1. ks-et1: Paired box gene 2 (*PAX2*), a pan-kidney cell epigenetic marker;
2. ks-et2: Gram domain containing 1B (*GRAMD1B*), a renal proximal tubular epithelial cell (RPTEC) and podocyte epigenetic marker; and
3. ks-et3: Dopa decarboxylase (*DDC*), a specific RPTEC epigenetic marker.

The ks-cfDNA assay was validated using plasma samples from a kidney transplant cohort [20]. Its performance for urinary ks-cfDNA was subsequently evaluated in an independent cardiac transplantation cohort comprising 10 cardiac transplant recipients who underwent serial postoperative urine sampling, including participants who developed postoperative kidney injury [21]. This cohort also included non-transplant comparator groups. Detailed clinical findings from this study will be reported separately [Murray et al., manuscript in preparation].

### Total cell-free DNA quantification

The beta-actin (*ACTB*) gene was used as a non-kidney specific positive control reflecting total cfDNA (t-cfDNA) and was targeted in a separate reaction. *ACTB* DNA is present in all nucleated cells, is understood to be biallelic and independent of methylation status. It has been widely used as a reference gene in other methylation assays due to its stable expression [22].

*ACTB* concentrations were used as a denominator to calculate the percentage of total cfDNA that was ks-cfDNA from an epigenetic target (%ks-et). Assays that had less than three positive partitions were considered below the lower limit of quantification.

### Sample collection, cfDNA extraction and ks-cfDNA quantification

Participants self-collected a mid-stream urine sample which was subsequently cooled to 4°C and centrifuged at 1600x*g* for 10 minutes as soon as practical. The time between collection and centrifugation of urine ranged from 5 minutes to 3 hours 30 minutes. Urine supernatant was aliquoted and at least 10mL of urine was stored at −80°C.

cfDNA was extracted from 4mL of urine using the QIAamp Circulating Nucleic Acid Kit and bisulfite converted using the EpiTect Fast Bisulfite Conversion Kit. 15μl of the volume eluant (equivalent to 1mL urine) was analysed by digital PCR (dPCR) using the QIAcuity Four (Qiagen) platform. Quantification of each ks-et was performed. Biomarker concentrations were normalised to urine creatinine. Laboratory personnel were not blinded to case/control status.

The principal laboratory procedures and kidney-specific methylation assays were consistent between the cardiac transplantation cohort and the present pregnancy cohorts. Urine collection and pre-analytical handling differed between the cardiac transplantation and present cohorts. In the cardiac transplantation cohort, urine was collected with Streck urine stabilisation reagent to permit delayed processing. In the present pregnancy cohort, participants provided self-collected mid-stream urine without cfDNA preservative but were centrifuged as soon as possible to minimise post-collection cellular lysis and cfDNA degradation.

### Statistical analysis

GraphPad Prism (version 10.0.2) was used for group comparisons and correlation analyses. Continuous variables were assessed for normality prior to analysis. Demographic variables were compared using an unpaired t-test with Welch’s correction, while categorical variables were compared using Fisher’s exact test. The Mann-Whitney U test was used to compare non-normally distributed values, including ks-et, t-cfDNA, %ks-et and urine creatinine.

Spearman’s rank correlation coefficient (ρ) was used to assess relationships between biomarkers, including correlations between ks-et biomarkers, t-cfDNA, urine creatinine, total urinary protein concentration, and available clinical variables.

Receiver operating characteristic (ROC) curves were constructed to evaluate the discriminatory performance of each biomarker and urine creatinine for PE. Area under the curve (AUC) values and 95% confidence intervals (CI) were calculated using the DeLong method. Optimal diagnostic thresholds were identified using Youden’s index.

To assess whether urine creatinine contributed independent discriminative ability beyond the ks-et biomarkers, a multivariable logistic regression model was fitted with PE as the binary outcome and ks-et1, ks-et2, and ks-et3 as covariates. A second model additionally included urine creatinine. The incremental value of urine creatinine was assessed using a likelihood ratio test comparing the two nested models. ROC and regression analyses were performed in R (version 4.6.0) using the pROC package for ROC analysis.

*P* values < 0.05 were considered significant.

## Results

### Participant characteristics

Participant characteristics are summarised in Table 1. There were no differences in mean gestational age (33.3 weeks in both groups) and nine PE participants had early PE (< 34 weeks). There was also no difference in maternal age (32.2 in NP compared with 34 years in PE) (*P* = 0.205). Women in the PE group had a significantly higher BMI at antenatal booking visit compared with the control group (31.6 compared with 24.4 kg/m^2^, *P* = 0.003). As expected, women with PE had a higher systolic blood pressure (SBP) (134 versus 112 mmHg) and diastolic blood pressure (DBP) (83 versus 71 mmHg) at recruitment compared with the NP participants (*P* < 0.001 for both SBP and DBP). Seven NP participants were nulliparous compared with 13 PE participants (*P* = 0.2*)*. No participants were undergoing induction of labour or were in labour at the time of sample collection.

**Table 1:** Participant characteristics.

	NP (*n* = 20)	PE (*n* = 20)	*P*
Age (years)	32.2 ± 3.4	34 ± 5.2	0.205
Gestation at recruitment (weeks)	33.3 ± 4.5	33.3 ± 4	0.987
Nulliparous	7	13	0.2
SBP (mmHg)	112 ± 8	134 ± 8.9	**< 0.001**
DBP (mmHg)	71.3 ± 6.7	82.9 ± 6.1	**< 0.001**
BMI at booking visit^1^ (kg/m^2^)	24.4 ± 7.3	31.6 ± 5.2	**0.003**
Participants with GDM (*n*)	4	8	0.301
Participants with pre-existing hypertension (*n*)	0	5	**0.047**
Participants with pre-existing CKD (*n*)	0	2	0.487
uPCR, as measured from urine sample (mg/mmol)^2^	–	36 (26.7–89.2)	
uPCR, from medical record^2^ (mg/mmol)	–	63 (33.6–82.3)	
Serum creatinine^2^ (μmol/L)	–	62.5 (50.5–70)	
PE with severe features (*n*)	–	6	
Early-onset PE (<34 weeks) (*n*)	–	9	
FGR at time of recruitment (*n*)	0	5	
Time from PE diagnosis to sample collection^3^ (days)	–	2 (2–8)	
Time from sample collection to centrifugation (mins)	84 (43–116)	63 (15–91)	0.324
Time from medical record uPCR to sample collection (days)	–	0 (0–0)	
Time from serum creatinine to sample collection^2^ (days)	–	0 (0–0)	
Time from sample collection to delivery (days)	14 (9–33)	10 (3–19)	0.079
Gestation at delivery^4^ (weeks)	37.5 ± 3.6	35.1 ± 3.8	0.057
SGA infant^5^ (*n*)	1	9	**0.008**

Values are expressed as the mean ± the standard deviation, or median (interquartile range). *P* values were calculated using an unpaired t test with Welch’s correction for continuous variables and a Fisher’s exact test for categorical variables.

SBP systolic blood pressure, DBP diastolic blood pressure, BMI body mass index, GDM gestational diabetes mellitus, CKD chronic kidney disease, uPCR urine protein-to-creatinine ratio, FGR fetal growth restriction [23], SGA small for gestational age [24].

^1^BMI at booking was not able to be calculated in 4 NP and 3 PE participants.

^2^Serum creatinine and uPCR data were not available for NP participants.

^3^Date of PE diagnosis not available for 1 participant.

^4^Gestation at delivery was not available for 3 NP and 1 PE participants.

^5^Birthweight not available for 3 NP and 1 PE participants.

When examining the differences in risk factors for the development of PE, five women in the PE cohort had pre-existing hypertension and two had underlying CKD. Eight women with PE had gestational diabetes mellitus (GDM) as opposed to four NP controls, but this was not statistically different between groups (*P* = 0.301). A single NP participant utilised assisted reproductive technology for conception and one woman in the NP cohort was a current smoker. No participants had pre-existing diabetes.

Most NP participants did not have a uPCR performed as part of clinical care. For the PE group the uPCR taken closest to the time of recruitment was retrieved from the medical record. Median uPCR, as recorded in the medical record, was 63 mg/mmol (interquartile range (IQR) 32.6–82.3). Urine samples collected from PE participants for ks-cfDNA analysis were also assessed for uPCR (mg/mmol) and total urinary protein concentration (g/L). The median uPCR from collected samples was 36 mg/mmol (IQR 26.7–89.2). Median serum creatinine (taken closest to the time of recruitment) in the PE cohort was 62.5 μmol/L (IQR 50.5–70). As seen in Fig 1, urine creatinine was significantly higher in the PE cohort (10.9 mmol/L (IQR 4.1–15.3)) compared to the NP group (3.9 mmol/L (IQR 2.3–5.6)) (*P* = 0.005).

**Fig 1:**
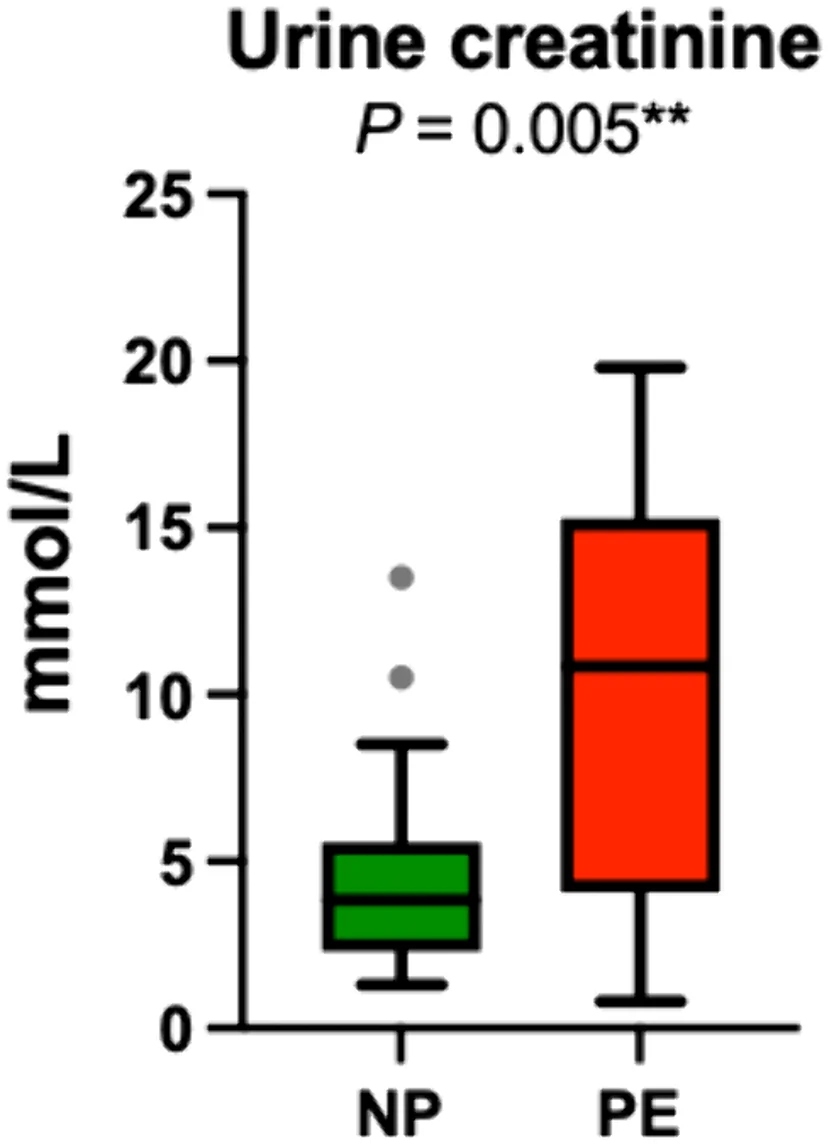
Comparison of urine creatinine between groups. Tukey box-and-whisker plot comparing urine creatinine (mmol/L) between normotensive pregnant (NP) and preeclamptic (PE) groups. Centre line denotes median value and box limits indicate 25^th^ and 75^th^ centiles. Whiskers extend to 1.5 x interquartile range (IQR). Points beyond whiskers represent outliers. Statistical significance was assessed using the Mann–Whitney U test. *P* value is shown.

Gestation at delivery was 37.5 ± 3.6 weeks in the NP group and 35.1 ± 3.8 weeks in the PE group (*P* = 0.057). When examining pregnancy outcomes a single NP participant had a small for gestational age infant compared with 9 PE participants (*P* = 0.008) [24]. A breakdown of medications in the PE cohort at recruitment and antihypertensive treatment at delivery is provided in S1 Table in S1 File. Details of labour, indications for delivery, and infant Apgar scores are provided in S2 Table in S1 File.

### Measurement of ks-cfDNA

Results are summarised in Fig 2. The concentrations of ks-et2 and ks-et3 were higher in PE participants. The median ks-et2 concentration in the PE group was 231 copies/mL (IQR 65–383) compared with 62 copies/mL (IQR 29–215) in the NP group (*P* = 0.028). Median ks-et3 was 81 copies/mL (IQR 28–199) for PE participants versus 20 copies/mL (IQR 9–81) for NP participants (*P* = 0.047). There was no significant difference in ks-et1 in PE (1485 copies/mL (IQR 260–2025) compared with NP 485 copies/mL (IQR 212–965, *P* = 0.052)).

**Fig 2:**
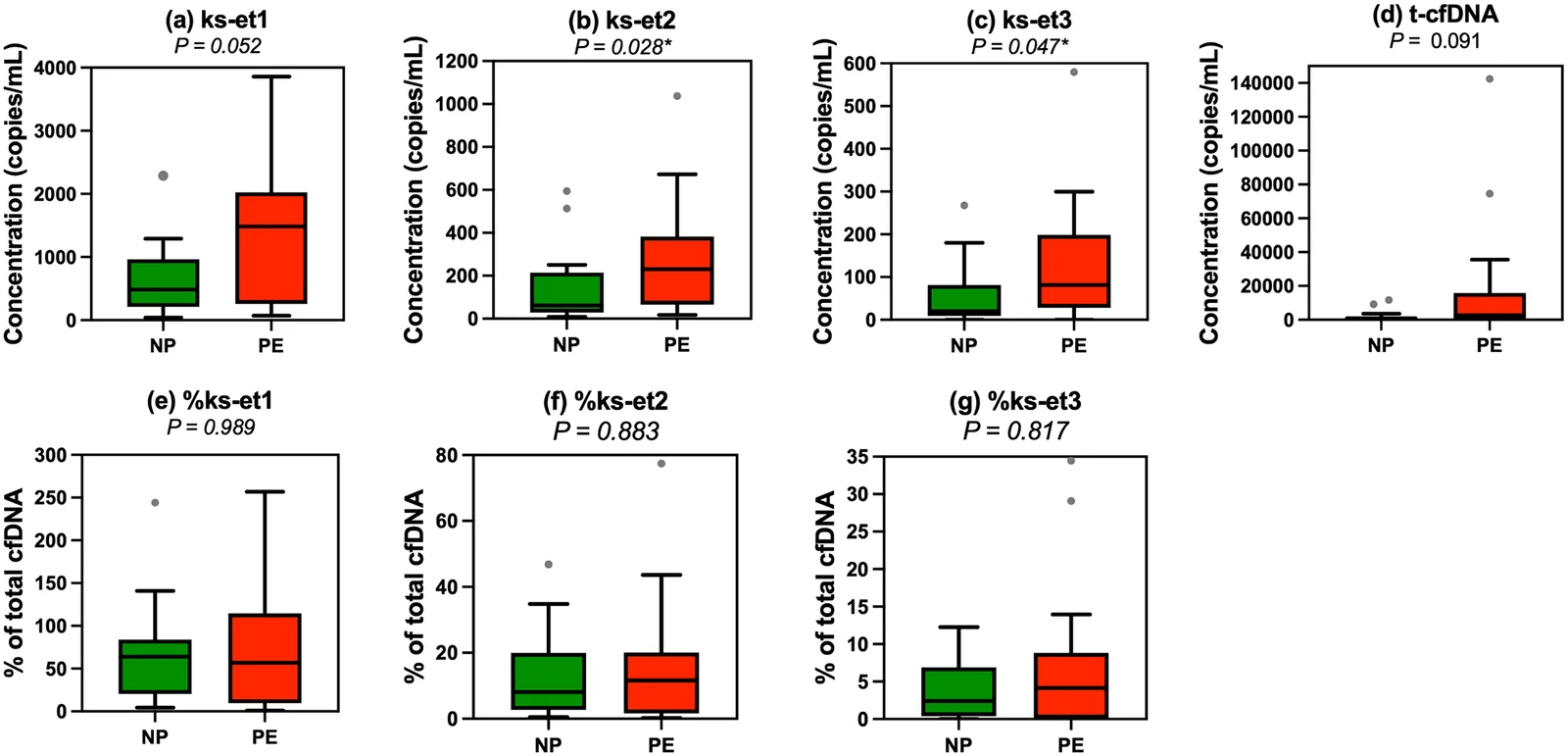
Comparison of ks-cfDNA quantities between groups. Tukey box-and-whisker plot comparing kidney-specific cell-free DNA (ks-cfDNA) between normotensive pregnant (NP) and preeclamptic (PE) groups. (a-c) Plots comparing the levels of urinary kidney-specific epigenetic targets (ks-et) and (d) total urinary cell-free DNA (t-cfDNA) between groups (copies/mL). (e-g) Each ks-et expressed as a percentage of t-cfDNA (%ks-et). Centre line denotes median value and box limits indicate 25^th^ and 75^th^ centiles. Whiskers extend to 1.5 x interquartile range (IQR). Points beyond whiskers represent outliers. Statistical significance was assessed using the Mann–Whitney U test. *P* values are shown.

Furthermore, there was higher concentration of t-cfDNA in the PE cohort (2697 copies/mL (IQR 601–15,843) in PE versus 914 copies/mL (IQR 431–1716) in NP), although this difference was not significant (*P* = 0.091).

When individual ks-et was expressed as a fraction of t-cfDNA (%ks-et), there was no difference between PE and NP cohorts for each methylation target (all *P* > 0.8) (Fig 2e–2g).

### Sensitivity analysis excluding PE participants with pre-existing hypertension

In a sensitivity analysis excluding the five PE participants with pre-existing hypertension, the differences in ks-et2 and ks-et3 were no longer statistically significant (ks-et2: 62 copies/mL [IQR 29–215] in NP versus 227 copies/mL [IQR 45–353] in PE, *P* = 0.093; ks-et3: 20 copies/mL [IQR 9–81] in NP versus 77 copies/mL [IQR 0–299] in PE, *P* = 0.174). Differences in ks-et1 and t-cfDNA remained non-significant (ks-et1: 485 copies/mL [IQR 212–965] in NP versus 1479 copies/mL [IQR 240–1750] in PE, *P* = 0.086; t-cfDNA: 914 copies/mL [IQR 431–1716] in NP versus 1212 copies/mL [IQR 512–3948] in PE, *P* = 0.240).

### Normalisation to urine creatinine

When the quantity of each ks-et, t-cfDNA and %ks-et was normalised to urine creatinine, there was no difference between PE and NP cohorts.

### Effect of urine concentration

As shown in Fig 3, urine creatinine demonstrated a moderate-to-strong correlation with ks-et1 (ρ = 0.7, *P* < 0.001), ks-et2 (ρ = 0.57, *P* < 0.001) and ks-et3 (ρ = 0.55, *P* < 0.001). In contrast, a weak correlation was observed between urine creatinine and t-cfDNA (ρ = 0.38, *P* = 0.015). Fractional measures (%ks-et) did not correlate with urine creatinine.

**Fig 3:**
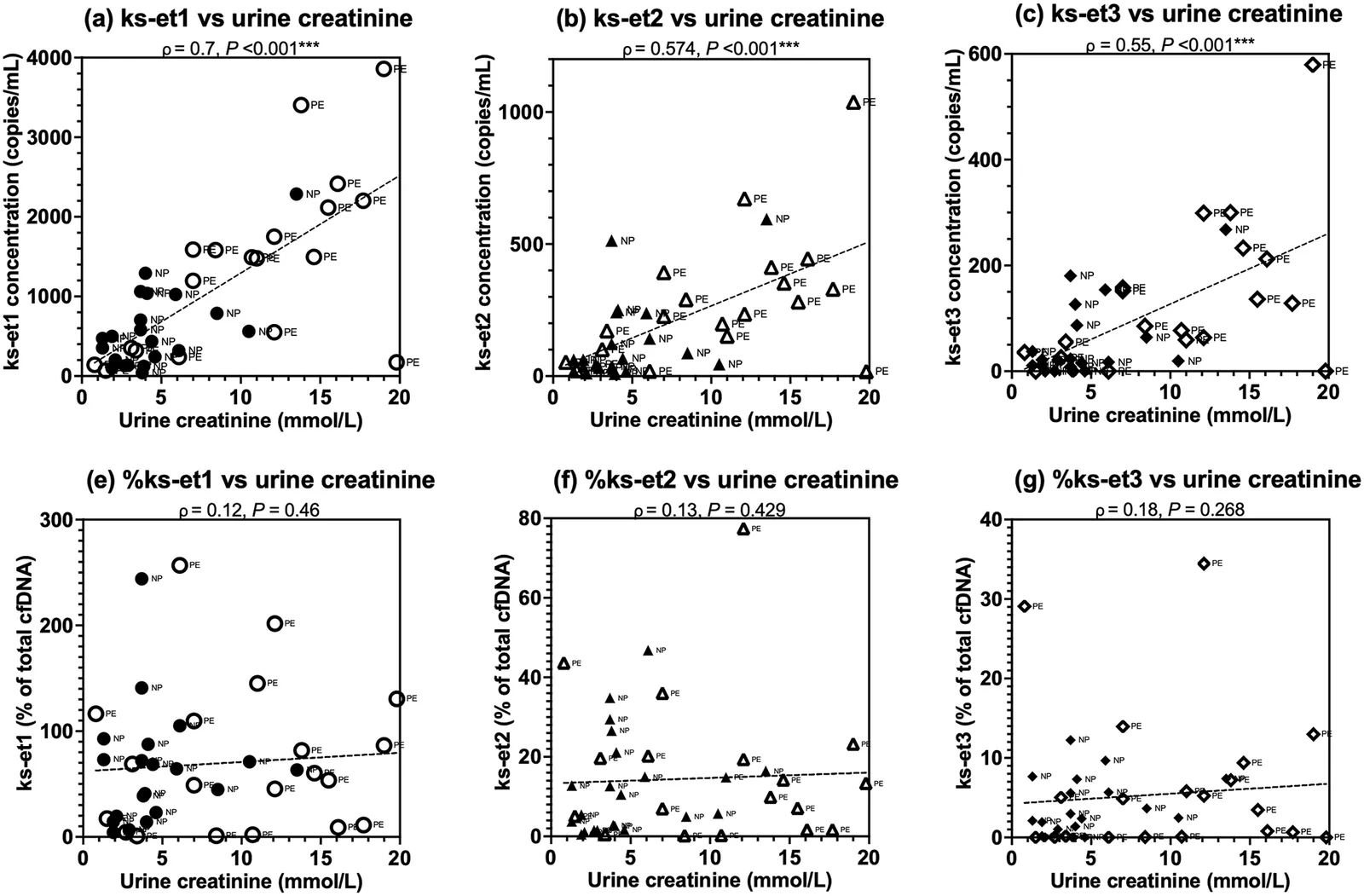
Correlation between ks-cfDNA and urine creatinine. Scatter plots showing relationships between urinary kidney-specific epigenetic targets (ks-et) and urine creatinine. (a-c) Each ks-et plotted against urine creatinine. A linear regression line is shown to illustrate the trend. (d) Urine creatinine versus total cell-free DNA (t-cfDNA), with t-cfDNA values plotted on a log_10_-scaled y-axis. (e-g) Fractional ks-et (%ks-et) plotted against urine creatinine. A linear regression line illustrates trend. Spearman’s correlation coefficient (ρ) and corresponding *P* values are shown for each plot. Each point represents an individual sample.

### Correlation between individual kidney-specific epigenetic targets

As seen in Table 2, all biomarkers increased proportionally with total cell-free DNA (ACTB) and each other, indicating general rather than selective elevation.

**Table 2:** Correlation between kidney-specific epigenetic targets and total cfDNA.

	ks-et1	ks-et2	ks-et3	t-cfDNA
ks-et1		ρ = 0.86 *P* < 0.001	ρ = 0.89 *P* < 0.001	ρ = 0.65 *P* < 0.001
ks-et2	ρ = 0.86 *P* < 0.001		ρ = 0.95 *P* < 0.001	ρ = 0.63 *P* < 0.001
ks-et3	ρ = 0.89 *P* < 0.001	ρ = 0.95 *P* < 0.001		ρ = 0.64 *P* < 0.001
t-cfDNA	ρ = 0.65 *P* < 0.001	ρ = 0.63 *P* < 0.001	ρ = 0.64 *P* < 0.001	

Spearman’s rank correlation coefficients (ρ) were used to compare quantities of kidney-specific epigenetic targets (ks-et) with one another and with total cell-free DNA (t-cfDNA).

### Discrimination of preeclampsia

ROC curves were plotted for each ks-et biomarker and urine creatinine (Fig 4). The AUC was 0.68 for ks-et1 (95% CI 0.5–0.86), 0.7 for ks-et2 (95% CI 0.54–0.87), and 0.68 for ks-et3 (95% CI 0.51–0.85). Urine creatinine demonstrated the highest AUC (0.76, 95% CI 0.59–0.92).

**Fig 4:**
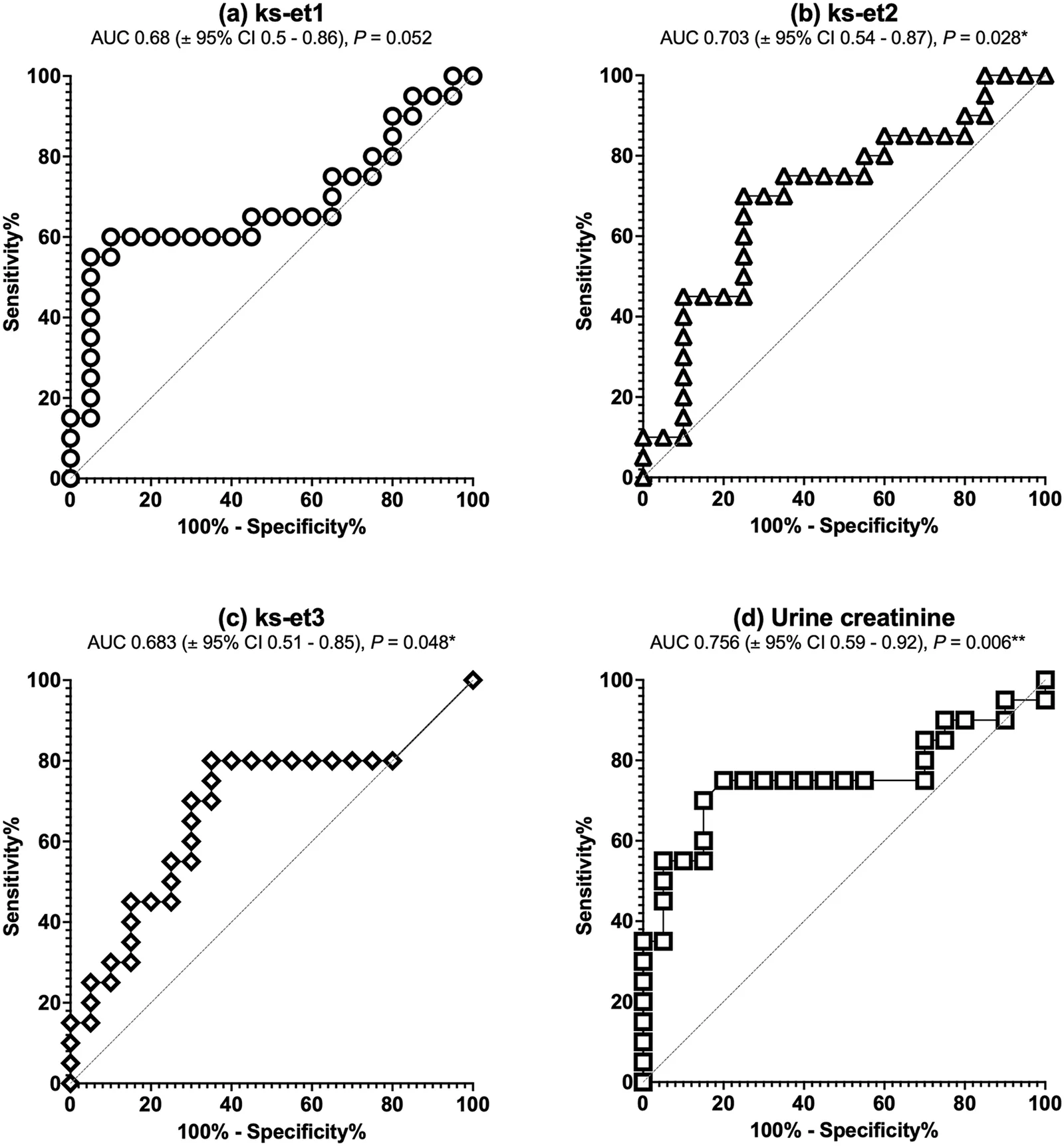
Receiver operating characteristic (ROC) curves for kidney-specific epigenetic targets and urine creatinine. Receiver operating characteristic (ROC) curves comparing kidney-specific epigenetic targets (ks-et) and urine creatinine between normotensive pregnant (NP) and preeclamptic (PE) groups. Area under the curve (AUC) values were calculated for: (a) ks-et1, (b) ks-et2, (c) ks-et3, and (d) urine creatinine**.** AUCs with 95% confidence intervals (CI) and corresponding *P* values are shown.

Comparison of AUC between urine creatinine and ks-et biomarkers using DeLong test showed no significant difference, likely reflecting limited power in this small cohort. On likelihood ratio testing, the addition of urine creatinine to the ks-et biomarkers improved model discrimination (χ² = 4.93, *P* = 0.026). In contrast, adding biomarkers to urine creatinine provided no additional predictive value (likelihood ratio test *P* > 0.6).

Youden Index analysis identified optimal diagnostic thresholds for each biomarker and is summarised in Table 3. ks-et1 demonstrated two equivalent optimal thresholds with sensitivity of 55–60% and specificity of 90–95% (*J* = 0.5). ks-et2 showed moderate performance, with an optimal cut-off corresponding to 70% sensitivity and 75% specificity (*J* = 0.45). ks-et3 demonstrated similar performance, with sensitivity of 80% and specificity of 65% (*J* = 0.45). Urine creatinine showed the highest overall performance, with sensitivity of 70–75%, specificity of 80–85%, and a maximum Youden Index of 0.55.

**Table 3:** Optimal diagnostic performance for kidney-specific epigenetic targets and urine creatinine.

Biomarker	Sensitivity (%)	Specificity (%)	Youden’s index (*J*)
ks-et1	55 - 60	90 - 95	0.5
ks-et2	70	75	0.45
ks-et3	80	65	0.45
Urine creatinine	70 - 75	80 - 85	0.55

Optimal diagnostic performance of kidney-specific epigenetic targets (ks-et) (ks-et1, ks-et2, ks-et3), and urine creatinine determined using Youden’s index. Sensitivity and specificity values correspond to the cut-off points that maximized Youden’s index for each biomarker. Higher Youden index values indicate better combined sensitivity and specificity for discriminating cases from controls.

### Association with proteinuria

Within PE cases, ks-et1 (but not ks-et2 or ks-et3) correlated with total urinary protein concentration (ρ = 0.47, *P* = 0.035) (Fig 5).

**Fig 5:**
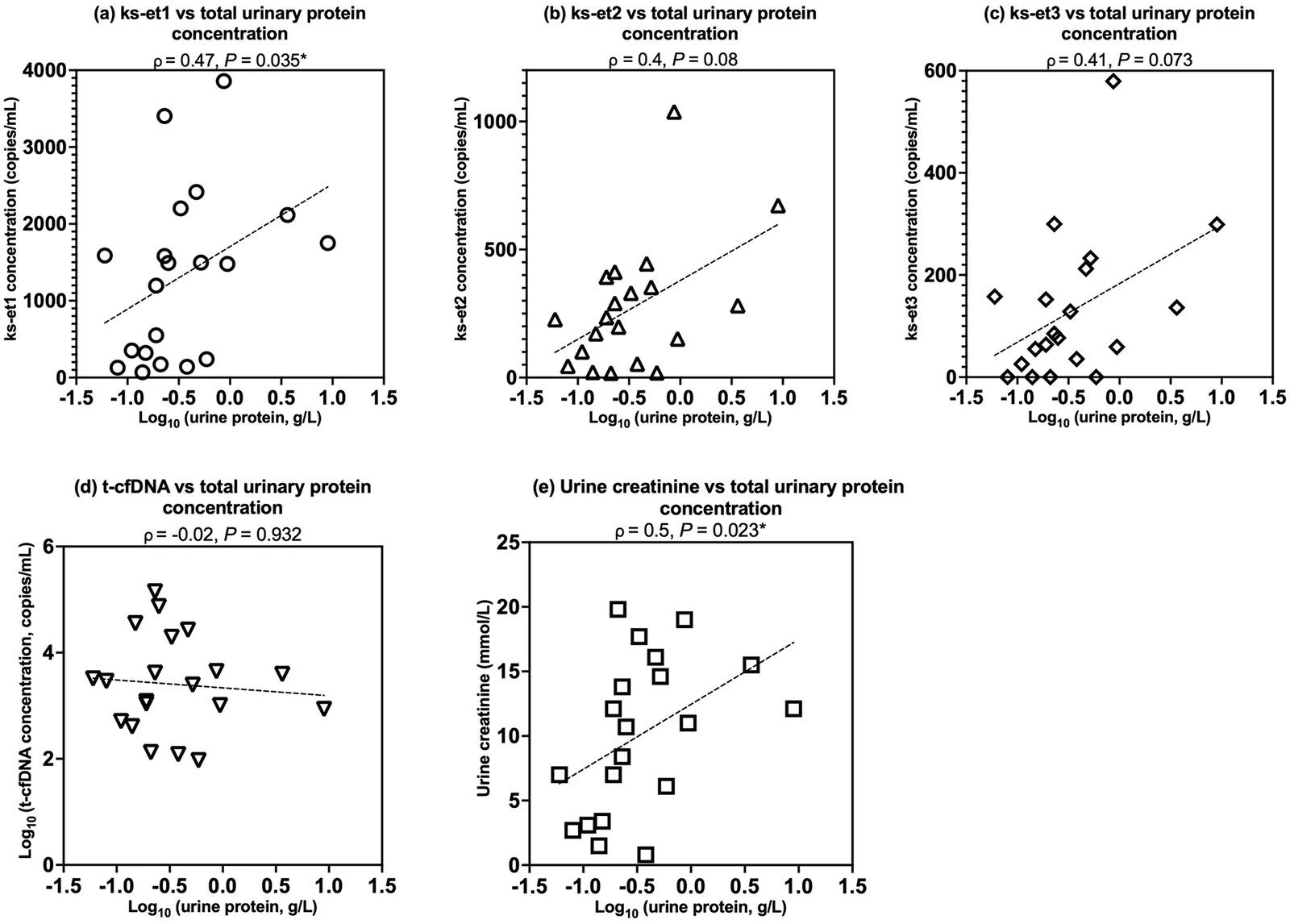
Correlation between kidney-specific epigenetic targets and total urinary protein concentration. Scatter plots showing (a-c) kidney-specific epigenetic targets (ks-et), (d) t-cfDNA, and (e) urine creatinine in relation to total urinary protein concentration (g/L) in preeclamptic (PE) cases. Total urinary protein concentration is displayed on the x-axis on a Log_10_ scale. A linear regression line is shown to illustrate the trend. Spearman’s rank correlation coefficients (ρ) and *P* values are provided for each plot. Each point represents an individual sample.

### Other clinical correlates

Given the difference in BMI between groups, an exploratory analysis was performed to assess the association between BMI at antenatal booking and t-cfDNA. No significant association was observed between BMI and t-cfDNA (ρ = 0.18, *P* = 0.312). Similarly, BMI was not significantly associated with ks-et or %ks-et quantities.

ks-et and %ks-et quantities did not correlate with serum creatinine in PE participants. Furthermore, quantities did not correlate with participant SBP and DBP readings or gestational age.

## Discussion

This exploratory study is the first to describe changes in urinary ks-cfDNA in women with PE. We hypothesised that PE would be associated with increased urinary ks-cfDNA, suggestive of kidney injury. The ks-cfDNA markers associated with RPTECs (ks-et2 and ks-et3) were elevated in PE compared with NP controls. ks-et2 is also expressed in podocytes.

This novel assay has also been studied in heart transplant patients, whereby ks-cfDNA (measured in blood and urine) allowed earlier detection of acute kidney injury [21]. Unlike transplantation, where genomic chimerism is used to distinguish donor and recipient DNA, this study used methylation patterns specific to kidney cells in a non-chimeric state.

These findings are consistent with previous work by You et al., who reported increased plasma ks-cfDNA, particularly kidney epithelial cell-derived cfDNA, in a group of participants with sepsis-induced kidney injury using genomic methylation sequencing. Notably, participants with sepsis not meeting criteria for kidney injury had increased levels of epithelial cell-derived cfDNA, suggesting that cfDNA changes may detect renal injury before conventional clinical measures [25]. Although the underlying condition, sample type and methodology differ, these findings support the potential of ks-cfDNA as a marker of renal injury.

The advantage of using urine as a disease biomarker is that it is a plentiful biological fluid, which can be non-invasively collected. However, physiological and pathological variation in urine composition is common, and may confound cfDNA assessments. Therefore, efforts were made to account for sources of variation in urine. Each ks-et was examined as a proportion of t-cfDNA (%ks-et) and urine creatinine was used to correct for urine concentration.

The elevation of urinary ks-cfDNA biomarkers in PE appeared attributable to increased urine concentration rather than cell subtype-specific injury. Kidney-derived cfDNA constitutes a substantial proportion of total urinary cfDNA, making absolute urinary cfDNA concentrations an informative measure of kidney-derived cfDNA release [26]. However, fractional measures (%ks-et) were also calculated to normalise each urinary ks-et to t-cfDNA. The lack of difference in fractional measures indicates that PE may be associated with a general increase in urinary ks-cfDNA release, rather than selective injury to cells associated with ks-et1, 2 or 3. Urine creatinine was higher in the PE group and the biological significance of this finding depends on whether elevated urine creatinine in PE reflects underlying renal pathophysiology, differences in tubular handling, or simply variation in urine concentration.

Increased urine creatinine has been attributed to changes in kidney function, creatine production and tubular creatinine secretion in preeclamptic individuals [27]. Urine creatinine is known to be affected by increased urinary protein excretion, and has been found to be elevated from as early as the first trimester in women who subsequently develop PE [27,28]. As proteinuria and PE are associated with physiologic changes in tubular function, elevated urine creatinine may not reliably reflect urine concentration in this population and may instead represent underlying renal pathophysiological changes in PE.

PE is associated with endothelial damage and podocyte injury mediated by alterations in angiogenic markers including vascular endothelial growth factor (VEGF), placental growth factor (PlGF) and soluble fms-like tyrosine kinase 1 (sFlt-1) [29–31]. However, PE is also associated with injury to vascular and tubular compartments of the kidney [32]. The observed trend toward increased ks-cfDNA associated with RPTECs is supported by studies by Wang et al. and Codsi et al. which demonstrated RPTEC injury in PE [33,34]. However, these findings should be interpreted cautiously in the absence of direct comparison with validated markers of tubular or glomerular injury. Comparison with established biomarkers such as urinary nephrin, podocin, kidney injury molecule-1 (KIM-1), neutrophil gelatinase-associated lipocalin (NGAL) and urinary albumin would be valuable in future studies. Regrettably, the ks-cfDNA epigenetic markers studied in this work did not incorporate endothelial cell-specific markers, and this remains an important avenue for further research. Additional appropriately selected methylation patterns may provide additional pathophysiological insight.

The ACTB gene (t-cfDNA) was used as a surrogate measure of total cfDNA in our study. Other studies have reported increased quantities of plasma total cfDNA levels in PE [9,35]. Our study found a non-significant trend towards t-cfDNA detected in the urine.

Given t-cfDNA was used to reflect total urinary cfDNA, t-cfDNA would be expected to exceed individual ks-et quantities, and this was mostly the case (median ks-et1 as a fraction of t-cfDNA was 61.77% (IQR 13.13–91.53)). However, in some cases it was observed that ks-et1 represented a large proportion, or was greater than t-cfDNA. t-cfDNA, measured using the ACTB gene, is run as a separate reaction to the ks-et assay so there is some variability in the readout. Increased ks-et1 compared to t-cfDNA likely reflects differences in assay amplification efficiency as opposed to biological changes. In future studies, calculation of the inter-assay coefficient of variation would help determine whether these differences are analytical rather than biological.

Hallmark histopathological features of preeclamptic nephropathy include glomerular endotheliosis together with podocyte injury and loss [36]. However, the absence of an endothelial-specific target is a limitation when applying this assay to PE. The lack of a significant increase in ks-et1, a pan-kidney marker, despite increased ks-et2 and ks-et3 was unexpected. This may reflect differences in assay amplification efficiency for the ks-et1 methylation target, as suggested by instances where ks-et1 exceeded t-cfDNA. Alternatively, the findings may reflect differences in the contribution of kidney cell types to urinary cfDNA. As ks-et2 captures both RPTEC and podocyte-specific methylation targets and endothelial-specific targets were not assessed, the relative contribution of individual renal compartments cannot be determined.

Notably, ks-et1 correlated with urinary total protein excretion. As proteinuria may reflect the extent of kidney involvement in PE [37], this association raises the possibility that ks-et1 may reflect broader nephron injury rather than compartment-specific damage alone. Therefore, ks-et1 warrants further investigation as a potential marker of kidney injury in PE through larger studies with more robust adjustment for potential confounders.

Obesity is a well-established risk factor for PE, and BMI was significantly higher in the PE cohort. Obesity has been linked to elevated circulating cfDNA levels, likely reflecting increased adipocyte turnover, cell necrosis and apoptosis, oxidative stress and low-grade inflammation [38–40]. There is also an established association between obesity and kidney injury [41]. In an exploratory analysis in the present cohort, BMI was not significantly associated with t-cfDNA. Nevertheless, the small sample size limits the ability to exclude residual confounding by BMI.

Furthermore, more women in the PE group had pre-existing hypertension, and circulating cfDNA has been associated with cardiometabolic risk factors, including hypertension [42]. By definition, women with PE have new-onset or worsening hypertension. In a sensitivity analysis excluding the five PE participants with pre-existing hypertension, differences in ks-et measures were no longer statistically significant. While hypertension is a potential confounder, the small sample size limits the interpretation of this sensitivity analysis and the extent to which its influence on the observed findings can be established. The impact of hypertension on urinary cfDNA has not been extensively studied. Medications administered to PE participants, including antihypertensive agents, may influence renal haemodynamics and also represent potential confounders. Given the limited cohort size, multivariable adjustment for important clinical confounders including BMI, pre-existing hypertension and medication use was not statistically robust and remains an important priority for future validation studies. Potential confounding by these clinical factors limits the ability of this study to establish whether the observed differences in ks-cfDNA are independently associated with PE in this cohort.

A further limitation of our study was that formal uPCR measures on the collected urine samples were lower than anticipated in the PE cohort (median uPCR 36 mg/mmol (IQR 26.66–89.21)). While proteinuria is not necessary for PE to be diagnosed [18], all but one PE participant had proteinuria (uPCR > 30 mg/mmol) documented prior to recruitment. Participants were recruited after diagnosis of PE, however, timing of recruitment in relation to diagnosis was not standardised, and clinical management before sample collection may have influenced the results. Some participants may have had lower uPCR results after initial diagnosis and following commencement of blood pressure lowering treatment prior to recruitment. However, the median time from the medical record uPCR to sample collection was zero days. Urinary measurements may also vary according to factors such as timing of collection and hydration status, while differences in laboratory handling between clinical and research samples may have contributed to variability. Nevertheless, this randomly selected cohort had reasonably low uPCR results which may reflect less pronounced kidney involvement. It would be worthwhile to determine whether results differed in a cohort with severe or early PE; however, our small sample size limited our ability to perform these sub-group analyses.

The relatively small sample size limited statistical power and resulted in wide confidence intervals around ROC estimates and biomarker performance metrics. Therefore, ROC analyses and Youden-derived thresholds should be considered hypothesis-generating and require validation in larger, independent cohorts. Although exploratory regression analyses were performed, the cohort size was insufficient for robust multivariable adjustment.

For ks-cfDNA to be further investigated as a potential biomarker in PE, these findings need to be validated in a larger cohort. The sFlt-1:PlGF ratio is the best available biomarker aiding in prediction and diagnosis of PE [43]. Correlating sFlt-1:PlGF results with ks-cfDNA markers would be useful in determining whether levels of ks-cfDNA correlate with angiogenic imbalance. We were unable to investigate this association as the sFlt-1:PlGF ratio was not widely used in this centre at time of recruitment. Longitudinal studies assessing urinary and plasma ks-cfDNA alongside the sFlt-1:PlGF ratio may further clarify the utility of ks-cfDNA for disease prediction, diagnosis and assessment of disease severity.

In summary, this is the first analysis of novel urinary ks-cfDNA biomarkers in PE and demonstrates the feasibility of this methodology. Preliminary differences in absolute concentrations of the RPTEC- and podocyte-specific ks-cfDNA biomarkers, ks-et2 and ks-et3, were observed in women with PE compared to NP controls. However, these findings should be interpreted cautiously given the influence of urinary concentration and the presence of pre-existing hypertension in some participants. Moreover, our data supports previous work by Kuromoto et al. (2010) identifying increased urine creatinine as a predictor of a PE diagnosis [27]. In this study urine creatinine outperformed ks-cfDNA in discriminating between preeclamptic and normotensive pregnancies. Whether urinary ks-cfDNA can provide additional clinical or pathophysiological insight beyond existing biomarkers requires confirmation in larger, longitudinal and appropriately adjusted studies.

## Supporting information

S1 Fig Comparison of absolute and fractional ks-cfDNA between groups. (a) Comparison of levels of kidney-specific epigenetic targets (ks-et) and total urinary cell-free DNA (t-cfDNA) in normotensive pregnant (NP) and preeclamptic (PE) groups (copies/mL). (b) Graph representing each ks-et as a fraction of t-cfDNA (%ks-et). y-axes plotted on a log_10_ scale. Median values outlined with error bars reflecting the interquartile range. Each point represents an individual sample.
(TIF)

S1 File Supplementary methods and tables. Supplementary methods and Tables S1 and S2.
(DOCX)
