# Supplementary material for: Analysis of urinary kidney-specific cell-free DNA in preeclampsia
Source: PLoS One. 2026 Sep 29;21(9):e0359105. doi: 10.1371/journal.pone.0359105 (PMC13623087; doi:10.1371/journal.pone.0359105)
Supplement: Supplementary file 1 [file pone.0359105.s002.docx]

# **Supporting information**

## **Supplementary methods**

The methylation targets were selected to be hypermethylated in the relevant renal tissues or cell types and minimally methylated in blood and other non-renal tissues. Tissue specificity was established using a DNA methylation atlas [20]. Assay sensitivity was evaluated by probit regression in accordance with CLSI EP17-A2. The PAX2 methylation assay achieved a 95% limit of detection corresponding to a kidney-derived DNA fraction of ≤ 0.625% at an input of 15 ng, while the more cell-restricted markers had higher detection limits. In contrived samples generated by spiking kidney genomic DNA into unrelated-donor plasma, measurements obtained using the PAX2 methylation assay showed strong agreement with an orthogonal genotype-based donor-derived cfDNA assay (R² = 0.978), supporting the specificity and quantitative performance of the methylation-based assay for kidney-derived DNA.

**S1 Table: Medications in the PE cohort at recruitment and delivery**

| **Antihypertensive medications at recruitment (*n*)** | PE (*n* = 20) |
| --- | --- |
| Labetalol  Metoprolol  Methyldopa  Nifedipine | 10  1  4  7 |
| **Number of antihypertensive medications at recruitment** |  |
| No antihypertensives  Single antihypertensive  Two antihypertensives  Three antihypertensives | 5  9  5  1 |
| **Other treatment prior to recruitment** |  |
| Antenatal corticosteroids | 9 |
| Magnesium sulfate | 0 |
| **Receiving antihypertensive medication at delivery** | 18 |

Values represent the number of participants. Antihypertensive medications, antenatal corticosteroid administration, and magnesium sulfate administration are reported at recruitment, while antihypertensive medication prescribed at delivery is reported separately. Participants may have received more than one antihypertensive medication at recruitment; therefore, individual medication counts do not sum to the total cohort.

**S2 Table: Labour, indication for delivery and mode of delivery**

|  | NP | PE |
| --- | --- | --- |
| **Labour (*n*)** | | |
| Spontaneous  Induction of labour  No labour | 9  3  5 | 0  7  12 |
| **Indication for delivery (*n*)** | | |
| Spontaneous  Planned delivery at term  Maternal indication  Fetal indication | 9  6  1  1 | 0  0  13  6 |
| **Mode of delivery** | | |
| Vaginal  Caesarean | 9  8 | 6  13 |
| **Apgar scores at delivery** | | |
| 1 minute Apgar score ≥7 (*n*)  5 minute Apgar score ≥7 (*n*) | 13  16 | 14  18 |

Values represent the number of participants. Maternal indication included planned delivery for preeclampsia at ≥ 37 weeks. Planned delivery at term included elective caesarean delivery or other planned delivery at term without a maternal or fetal indication.

Labour, indication for delivery and mode of delivery data were unavailable for 3 NP and 1 PE participants. Apgar scores were missing for 3 NP and 2 PE participants.
